# Autologous tolerogenic dendritic cells for rheumatoid and inflammatory arthritis

**DOI:** 10.1136/annrheumdis-2015-208456

**Published:** 2016-04-26

**Authors:** G M Bell, A E Anderson, J Diboll, R Reece, O Eltherington, R A Harry, T Fouweather, C MacDonald, T Chadwick, E McColl, J Dunn, A M Dickinson, C M U Hilkens, John D Isaacs

**Affiliations:** 1 Arthritis Research UK Rheumatoid Arthritis Pathogenesis Centre of Excellence (RACE), Musculoskeletal Research Group, Institute of Cellular Medicine, Newcastle upon Tyne, UK; 2 Institute of Health and Society, Faculty of Medical Sciences, Newcastle University, Newcastle upon Tyne, UK; 3 Clinical Trials Unit, Faculty of Medical Sciences, Newcastle University, Newcastle upon Tyne, UK; 4 Haematological Sciences, Institute of Cellular Medicine, Newcastle upon Tyne, UK

**Keywords:** Rheumatoid Arthritis, Treatment, Synovial fluid, Inflammation

## Abstract

**Objectives:**

To assess the safety of intra-articular (IA) autologous tolerogenic dendritic cells (tolDC) in patients with inflammatory arthritis and an inflamed knee; to assess the feasibility and acceptability of the approach and to assess potential effects on local and systemic disease activities.

**Methods:**

An unblinded, randomised, controlled, dose escalation Phase I trial. TolDC were differentiated from CD14+ monocytes and loaded with autologous synovial fluid as a source of autoantigens. Cohorts of three participants received 1×10^6^, 3×10^6^ or 10×10^6^ tolDC arthroscopically following saline irrigation of an inflamed (target) knee. Control participants received saline irrigation only. Primary outcome was flare of disease in the target knee within 5 days of treatment. Feasibility was assessed by successful tolDC manufacture and acceptability via patient questionnaire. Potential effects on disease activity were assessed by arthroscopic synovitis score, disease activity score (DAS)28 and Health Assessment Questionnaire (HAQ). Immunomodulatory effects were sought in peripheral blood.

**Results:**

There were no target knee flares within 5 days of treatment. At day 14, arthroscopic synovitis was present in all participants except for one who received 10×10^6^ tolDC; a further participant in this cohort declined day 14 arthroscopy because symptoms had remitted; both remained stable throughout 91 days of observation. There were no trends in DAS28 or HAQ score or consistent immunomodulatory effects in peripheral blood. 9 of 10 manufactured products met quality control release criteria; acceptability of the protocol by participants was high.

**Conclusion:**

IA tolDC therapy appears safe, feasible and acceptable. Knee symptoms stabilised in two patients who received 10×10^6^ tolDC but no systemic clinical or immunomodulatory effects were detectable.

**Trial registration number:**

NCT01352858.

## Introduction

Despite major therapeutic advances, there is no cure for rheumatoid arthritis (RA) and only a small proportion of patients achieve drug-free remission, which is often transient.^1^ For the remainder, the need for chronic medications with associated side effects, sometimes serious, impacts on overall quality of life. The ideal management of RA is a therapy that returns the immune system to a state of self-tolerance, reversing autoimmunity without requiring long-term treatment.

Dendritic cells (DCs) orchestrate immune responses, by ingesting and presenting antigens to T cells.^2^ ^3^ In health they direct immune attacks against pathogens and tumours and, in a distinct state of differentiation, play an important role in maintaining self-tolerance.^4^ ^5^ In contrast, in autoimmunity DCs drive activation and differentiation of autoreactive effector T cells.^6^ If this inappropriate activation could be reversed and immune regulation restored, self-tolerance should re-emerge.

Over the past 10 years, we have developed a method to differentiate human tolerogenic DC (tolDC) from the blood of healthy individuals and patients with inflammatory arthritis.^7–10^ Unlike conventional mature DCs, which produce interleukin (IL)-12p70 and other proinflammatory cytokines, tolDC produce no IL-12p70 but high levels of IL-10. They deviate naïve T cells towards an IL-10-producing phenotype and induce hyporesponsiveness in memory T cells. Importantly, in mixed cultures they dominate mature, proinflammatory DCs and downregulate T-cell activation. Their phenotype is stable in the presence of proinflammatory stimuli. Equivalent murine tolDC switch off collagen-induced arthritis (CIA), with immune deviation from IL-17 to IL-10 production by CD4+ T cells and a reduction in type II collagen-specific T-cell responses.^11^ While our data implicate IL-10 as a key anti-inflammatory cytokine, it can also boost B-cell responses with pro-immune effects.^12^

We now report the results of a Phase I trial of autologous tolDC in patients with rheumatoid and inflammatory arthritis. This is only the second reported trial of tolDC in inflammatory arthritis^13^ and the first to use an intra-articular (IA) route of administration, chosen to optimise the detection and management of potential AEs.

## Methods

### TolDC manufacture

We previously reported our method for manufacturing therapeutic grade tolDC investigational medical product (IMP).^9^ Our full good manufacturing practice (GMP) protocol is provided in the online supplementary methods. TolDC were loaded with autologous synovial fluid (SF) as a source of relevant autoantigens,^14–16^ enabling the treatment of both patients with seropositive RA and patients with seronegative RA, as well as other arthritides. Prior to administration, tolDC satisfied all quality control (QC) release criteria (table 1).

**Table 1:** Summary of QC release criteria data

			Purity		Phenotype			Functionality			
Patient	Dose	Viability (%)*	CD11c (%)‡	HLA-DR (%)‡	CD83 (MFI)§	CD86 (MFI)¶	TLR2 (MFI)**	IL-10 (pg/mL)††	IL-12 (pg/mL)‡‡	Sterility†	QC result
1	1×10^6^	88	100	100	58	462	1434	2364	<50	Pass	Pass
2	Placebo	–	–	–	–	–	–	–	–	–	–
3	1×10^6^	88	99.9	100	41	773	1188	3373	<50	Pass	Pass
4	1×10^6^	90	100	100	109	356	758	10 510	<50	Pass	Pass
5	3×10^6^	90	99.9	100	173	384	2085	2295	<50	Pass	Pass
6	Placebo	–	–	–	–	–	–	–	–	–	–
7	3×10^6^	96	100	99.9	73	390	2921	5164	<50	Pass	Pass
8	3×10^6^	96	100	100	61	1878	251	1331	<50	Pass	Fail
9	3×10^6^	71	100	100	70	772	926	2040	<50	Pass	Pass
10	10×10^6^	80	100	100	83	264	4276	2425	<50	Pass	Pass
11	Placebo	–	–	–	–	–	–	–	–	–	–
12	10×10^6^	79	100	100	62	464	1704	4679	<50	Pass	Pass
13	10×10^6^	95	99.8	100	135	320	1473	1607	<50	Pass	Pass

*Viability was determined using trypan blue exclusion and expressed as percentage of total cells. To pass quality control (QC) viability had to be >70%.

†Sterility was assessed by BacT/Alert and fungal screen on day 0, day 3 and final product (day 7). To pass QC, the product had to pass sterility screening with no organisms detected.

‡CD11c and human leucocyte antigen—antigen D-related (HLA-DR) expression determined using flow cytometry and expressed as a percentage of viable cells. To pass QC, the percentage of positive cells had to be >90% for both markers.

§CD83 expression determined using flow cytometry and expressed as a median fluorescence intensity (MFI) of viable cells. To pass QC, CD83 MFI had to be <350.

¶CD86 expression determined using flow cytometry and expressed as a MFI of viable cells. To pass QC, CD86 MFI had to be <1500.

**Toll-like receptor (TLR)2 expression determined using flow cytometry and expressed as a MFI of viable cells. For information only. Positive result=TLR2 MFI >200.

††Interleukin (IL)-10 levels in supernatant from the 7 days culture were determined by ELISA. To pass QC, IL-10 levels had to be >1000 pg/mL.

‡‡IL-12 levels in supernatant from the 7 days culture were determined by ELISA. To pass QC, IL-12 levels had to be <50 pg/mL.

10.1136/annrheumdis-2015-208456.supp1 Supplementary data

### Participants

Participants, aged 18 or over, had inflammatory arthritis of at least 6 months' duration, including an inflamed knee joint with an effusion and at least 30 min early morning stiffness. They had failed at least one disease-modifying antirheumatic drug (DMARD), including current therapy. TolDC was added to stable DMARD and anti-inflammatory therapies. Intramuscular glucocorticoids and IA injections of the target knee were not permitted for 6 weeks prior to baseline. Standard exclusion criteria were applied (see online supplementary table S1).

10.1136/annrheumdis-2015-208456.supp2 Supplementary data

### Study design

This was an unblinded, randomised, controlled, dose escalation Phase I trial of IA tolDC administered into an inflamed knee joint (the target knee). The trial protocol, available at http://clinicaltrials.gov/ct2/show/NCT01352858, was approved by the Medicines and Healthcare Products Regulatory Agency and by the National Research Ethics Service Committee North East (Sunderland) (EudraCT number: 2011-001582-41). The trial was conducted according to the International Council for Harmonisation of Technical Requirements for Pharmaceuticals for Human Use Good Clinical Practice (ICH GCP) and the Declaration of Helsinki. There were three dosing cohorts of 1×10^6^, 3×10^6^ and 10×10^6^ viable tolDC administered via a single arthroscopic injection following saline irrigation. Each cohort comprised four participants, randomly allocated to tolDC (n=3) or control intervention of target knee arthroscopic saline irrigation only (n=1). The decision to dose escalate between cohorts was decided by an independent data-monitoring committee based on safety and tolerability data, recorded 5 days after treatment of the last participant in each cohort.

**Figure 1:**
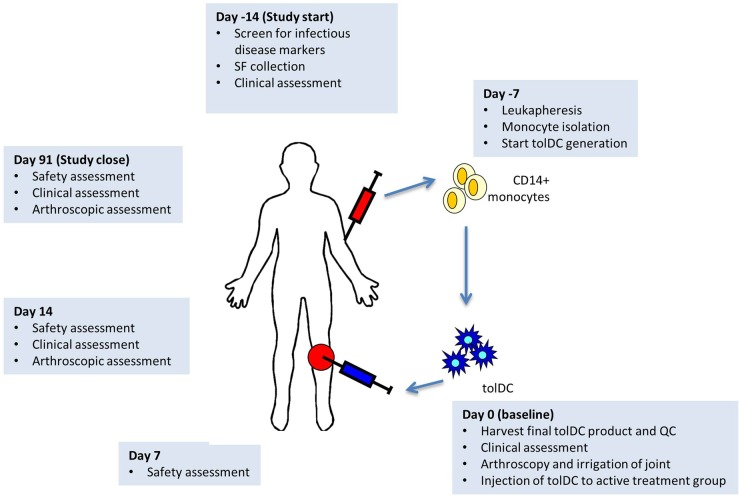
Overview showing tolerogenic dendritic cells (tolDC) treatment protocol. Following informed consent on day −14, an infectious disease screen was performed and synovial fluid (SF) aspirated under ultrasound guidance for use during tolDC manufacture. Participants returned 7 days later (day −7) for leucapheresis, their leucapheresis product being transferred to the good manufacturing practice facility for initiation of tolDC manufacture. After a further 7 days participants returned (day 0, baseline visit) and, following a clinical assessment and ultrasound assessment of the target knee, underwent fibre-optic arthroscopy. The target knee joint was irrigated with 1 L of normal saline, following which tolDC were administered arthroscopically. On days 7 and 14 participants returned for safety assessments. The day 14 visit also entailed an arthroscopic assessment of the target knee. If the participant remained symptomatic and/or the knee remained inflamed an arthroscopic intra-articular glucocorticoid injection was administered. The final study visit on day 91 was identical to the day +14 visit.

The study design is outlined in figure 1. Following informed consent on day −14, an infectious disease screen was performed and SF aspirated for use during tolDC manufacture. Participants returned on day −7 for leucapheresis. At the baseline visit (day 0), the target joint was arthroscopically irrigated followed by tolDC administration. IA administration was chosen to provide an early and robust signal of disease deterioration and the opportunity for joint irrigation under those circumstances.

### Primary outcome

The primary objective of Autologous Tolerogenic Dendritic Cells for Rheumatoid and Inflammatory Arthritis (AuToDeCRA) was to assess safety of the intervention. Participants were questioned about symptomatic deterioration by telephone on days 1–5, with particular focus on the target knee. If deterioration was reported on two successive days, participants were assessed in person. If deterioration was confirmed, a further arthroscopic examination was performed with irrigation and IA glucocorticoid if indicated. If infection was suspected, this was managed appropriately. Knee assessment tools standardised subjective and objective assessments (see online supplementary figures S1 and S2). Routine safety assessments took place on days 7 and 14. The latter included a further arthroscopic examination and, if synovitis persisted, an arthroscopic IA glucocorticoid injection was administered. The final study visit on day 91 was identical to day 14, except arthroscopy was only indicated if patients had not previously received IA glucocorticoid during the study.

The primary outcome was the proportion of participants experiencing a target knee flare within 5 days of tolDC administration and, additionally, the proportion experiencing serious AEs (SAEs) and AEs throughout the trial. Although tolDC were stable in vitro, a significant concern was their potential to become activated in an inflamed environment—our experiments in murine CIA demonstrated worsening of joint inflammation when antigen-loaded mature DCs were administered.^11^ Knee flares beyond 5 days were deemed more likely to represent inflammation returning post-irrigation, whereas, if tolDC were efficacious, we predicted prolonged symptomatic benefit.

### Secondary and exploratory outcomes

Secondary objectives were to assess feasibility and tolerability. Feasibility was defined by the proportion of participants entering the study from whom tolDC could be prepared (the success rate of tolDC manufacture). Tolerability was scored as the proportion of participants who rated the study and its components as partly or completely acceptable, assessed via a questionnaire administered at the final study visit (see online supplementary figure S3).

Exploratory objectives included assessment of the potential effects of tolDC on local and systemic disease activities. Exploratory outcomes included arthroscopic assessment of target knee synovitis (days 0 and +14); disease activity score (DAS)28 and Health Assessment Questionnaire (HAQ) at each study visit; peripheral blood T-cell phenotype assessed by intracellular cytokine staining and peripheral blood cytokine levels (days 0, 14 and 91, see online supplementary methods). Arthroscopy was performed by an unblinded investigator using a published method.^17^

### Statistics

This Phase I trial was not powered for comparative hypothesis testing and basic descriptive statistics are used to summarise outcome data, demographic and operational information.

## Results

### Participants

Sixteen participants were screened. SF could not be obtained from three and the cell product did not meet release criteria in participant 8 (see below). Of the remaining 12 participants, 6 had seropositive RA, 1 had seronegative RA, 3 had psoriatic arthritis and 2 had undifferentiated seronegative arthritis (table 2). The 10×10^6^ tolDC cohort contained only one patient with RA, whereas the lower dose cohorts contained three each. Disease duration ranged from 2 to 43 years and DAS28 ranged from 1.4 to 6.0. Background DMARD therapy ranged from nil to biological therapy.

**Table 2:** Participant demographics, current and prior treatment and experimental cohort

Participant number	Age (years)	Diagnosis	Disease duration (years)	DAS28 at baseline	Background DMARD treatment	Prior treatments	tolDC treatment
1	75	Seropositive RA	43	2.6*	PEN	Nil	1×10^6^
2	59	Seropositive RA	19	4.5	MTX s/c	SSZ, po MTX	Washout
3	53	Seropositive RA	14	6.0	ADA	PEN, gold, SSZ, MTX, LEF, ETA, ADA, RTX	1×10^6^
4	56	Psoriatic arthritis	6	2.6	MTX, LEF	Nil	1×10^6^
5	41	Undifferentiated seronegative arthritis	3	3.5*	MTX	SSZ	3×10^6^
6	63	Seropositive RA	31	5.1	MTX, ETA	MTX, LEF, gold, SSZ, PEN	Washout
7	35	Seropositive RA	3	4.4	MTX, SSZ, HCQ	Nil	3×10^6^
8†	47	Seronegative arthritis	1	1.4	MTX	SSZ	3×10^6^†
9	60	Seronegative RA	14	5.2	MTX, HCQ	Nil	3×10^6^
10	77	Psoriatic arthritis	3	4.4*	MTX, SSZ	Nil	10×10^6^
11	65	Seropositive RA	2	4.5	MTX, SSZ, HCQ	Nil	Washout
12	57	Undifferentiated seronegative arthritis	4	2.3	MTX	Nil	10×10^6^
13	45	Psoriatic arthritis	18	2.2	Nil	MTX, SSZ	10×10^6^

*Screening DAS28 values.

†Cells failed release criteria.

ADA, adalimumab; DMARD, disease-modifying antirheumatic drug; ETA, etanercept; HCQ, hydroxychloroquine; LEF, leflunomide; MTX, methotrexate; PEN, penicillamine; po, oral administration; RA, rheumatoid arthritis; RTX, rituximab; s/c, subcutaneous administration; SSZ, sulfasalazine; tolDC, tolerogenic dendritic cells.

### Product characteristics

Table 1 provides the QC release criteria for tolDC, encompassing viability, sterility, phenotype and function. The IMP generated from cells of participant 8 had higher cell surface CD86 expression than specified in our release criteria and therefore could not be released as part of the clinical trial. All other release criteria were met and, following informed discussion, the participant elected to receive the product but their data are reported separately. Toll-like receptor 2 is upregulated during tolDC differentiation and, while recorded in table 1, did not constitute a release criterion. Online supplementary figure S4 exemplifies flow cytometry QC data.

### Primary outcome and AEs

No participants developed worsening symptoms in the target knee during days 1–5. There were two SAEs, both in participant 3 with highly active, refractory RA (table 3). A generalised RA flare occurred on day 70, requiring hospitalisation. Adalimumab was switched to tocilizumab but the participant was re-admitted 15 days later with pneumonia requiring IV antibiotics. Both events were felt unlikely to be related to tolDC.

**Table 3:** Adverse events and serious adverse events (SAEs)

Study number	Cohort	Adverse event	Grade	Day	Action	Relationship to treatment	Relationship to procedure
1	1×10^6^ tolDC	Discomfort right heel	Mild	0	Nil	Unrelated	Probable (secondary to immobilisation)
1	1×10^6^ tolDC	Redness right heel	Mild	0	Nil	Unrelated	Probable (secondary to immobilisation)
1	1×10^6^ tolDC	Bruising below knee	Mild	1	Nil	Unrelated	Related to arthroscopy
1	1×10^6^ tolDC	Target knee synovitis	Moderate	10	Aspiration d10, IA glucocorticoid d14	Possible	Unrelated
1	1×10^6^ tolDC	Wound infection target knee	Mild	31	Oral flucloxacillin	Unlikely	Related to arthroscopy
2	Control	Leg cramps	Mild	≈14	Quinine sulfate	Unrelated	Unlikely
3	1×10^6^ tolDC	Iron deficiency anaemia	Moderate	−14	Ferrous sulfate	Unrelated	Unrelated
3	1×10^6^ tolDC	Citrate Toxicity	Mild	−7	Nil	Unrelated	Related to leucapheresis
3	1×10^6^ tolDC	Increased target knee pain	Moderate	0	Analgesia	Unlikely	Related to arthroscopy
3	1×10^6^ tolDC	Fatigue	Mild	1	Nil	Unlikely	Possible
3	1×10^6^ tolDC	General RA flare	Moderate	9	IM glucocorticoid	Possible	Unrelated
3	1×10^6^ tolDC	General RA flare	N/A (SAE)	70	Hospitalised, commenced tocilizumab	Unlikely	Unrelated
3	1×10^6^ tolDC	Pneumonia	N/A (SAE)	85	Hospitalised, antibiotics	Unlikely	Unrelated
4	1×10^6^ tolDC	Increased stiffness of target knee	Moderate	1	Nil	Unlikely	Possible
4	1×10^6^ tolDC	New patch of psoriasis on forearm	Mild	6	Topical steroid/calcipitriol	Possible	Unrelated
4	1×10^6^ tolDC	Increased pain both knees	Mild	6	Nil	Possible	Unrelated
5	3×10^6^ tolDC	Flare IA	Mild	−7	Nil	Unrelated	Unrelated
5	3×10^6^ tolDC	Rhinorrhoea	Mild	3	Nil	Possible	Unrelated
5	3×10^6^ tolDC	Target knee synovitis	Moderate	10	Aspirated, naproxen dose ↑	Possible	Unrelated
5	3×10^6^ tolDC	Eczema right elbow	Mild	57	Topical hydrocortisone	Unlikely	Unrelated
6	Control	Rash right forearm	Mild	−7	Nil	Unrelated	Unrelated
6	Control	Upper respiratory tract infection	Mild	44	Oral amoxicillin	Unrelated	Unrelated
7	3×10^6^ tolDC	Increased stiffness in target knee	Mild	9	Ibuprofen	Possible	Unrelated
8*		Wound infection	Mild	2	Oral flucloxacillin	Unrelated	Related to arthroscopy
9	3×10^6^ tolDC	Non-target knee synovitis	Moderate	0	Ibuprofen	Unrelated	Unrelated
9	3×10^6^ tolDC	Fatigue	Mild	4	Nil	Possible	Unrelated
9	3×10^6^ tolDC	Elevated C reactive protein and bilateral knee synovitits	Moderate	7	IA glucocorticoid	Possible	Unrelated
9	3×10^6^ tolDC	Fatigue	Mild	10	Nil	Possible	Unrelated
9	3×10^6^ tolDC	Fluid leak from target knee wound	Mild	21	Oral flucloxacillin	Unrelated	Related to arthroscopy
10	10×10^6^ tolDC	Increased target knee pain after long walk	Mild	6	Nil	Possible	Unrelated
10	10×10^6^ tolDC	Worsening psoriasis bottom of feet	Mild	55	Topical calcipitriol	Possible	Unrelated
10	10×10^6^ tolDC	Arthralgia due to osteoarthritis	Mild	≈60	Nil	Unrelated	Unrelated
11	Control	Iron deficiency anaemia	Moderate	≈11	Ferrous gluconate	Unlikely	Possible
11	Control	Rhinorrhoea	Mild	13	Nil	Unrelated	Unrelated
13	10×10^6^ tolDC	Vasovagal episode	Mild	0	Nil	Unrelated	Related to arthroscopy
13	10×10^6^ tolDC	Upper respiratory tract infection	Mild	11	Paracetamol	Possible	Unrelated
13	10×10^6^ tolDC	General stiffness and discomfort	Mild	23	Nil	Possible	Unrelated
13	10×10^6^ tolDC	Folliculitis	Mild	28	Nil	Possible	Unrelated
13	10×10^6^ tolDC	Swelling non-target knee with effusion	Mild	84	IA glucocorticoid	Unlikely	Unrelated

*Cells from study number (participant) 8 did not meet QC release criteria, see table 1.

IA, intra-articular; IM, intra-muscular; N/A, not applicable; RA, rheumatoid arthritis; tolDC, tolerogenic dendritic cells.

37 AEs were recorded (table 3). 15 were felt possibly related to therapy, largely because of their timing. Despite the lack of protocol-defined target knee flares, there were three episodes of target knee synovitis requiring treatment, noted on days 7 (participant 9) and 10 (participants 1 and 5). Participant 9 also had contralateral knee synovitis, present since baseline. In addition participant 3, who was hospitalised with an RA flare on day 70, also suffered a flare on day 9. Two of these AEs occurred in the 1×10^6^ tolDC and two in the 3×10^6^ tolDC cohort. Participant 7 reported increased target knee stiffness on day 9 and participant 13 reported generalised stiffness and discomfort on day 23 but, clinically, these were not disease flares. Participant 13 subsequently developed non-target knee synovitis on day 84. Two episodes of rhinorrhoea (3×10^6^ tolDC and control), two episodes of upper respiratory tract infection (10×10^6^ tolDC and control) and folliculitis (10×10^6^ tolDC) were the only infectious AEs, excluding wound infections (see below). Two participants with psoriatic arthritis reported minor worsening of psoriasis on days 6 and 62. There were two reports of self-resolving knee pain, one provoked by exercise.

Twelve AEs were possibly, probably or definitely attributable to procedures, including two wound infections (one in participant 8 whose product failed QC) and a fluid leak, an episode of citrate toxicity related to leucapheresis and a vasovagal episode related to arthroscopy. Self-resolving AEs occurring within 24 h of tolDC administration, such as fatigue or target knee pain or stiffness, were attributed to the procedure rather than to tolDC. All AEs were assessed as mild or moderate with no evidence of a dose–response relationship. In particular knee, or systemic disease, flares only occurred in lower dose cohorts, apart from a late (day 84) non-target knee flare in participant 13.

### Secondary outcomes—feasibility and participant acceptability

TolDC that met release criteria were manufactured from 9 of 10 production runs (see above). Participant acceptability was high (table 4). About 91% of participants rated the study overall as acceptable. Equivalent percentages were 88%, 75%, 91% and 64% for leucapheresis, knee aspiration, ultrasound and arthroscopy. About 91% found participation convenient and 90% would participate again.

**Table 4:** Participant acceptability scores

Question	N	1. Totally disagree	2. Partly Disagree	3. Neither agree nor disagree	4. Partially agree	5. Totally agree	Per cent answer 4 or 5	Per cent answer 5
Taking part in the study was convenient	11	1	0	0	2	8	91	73
The overall study was acceptable	11	1	0	0	1	9	91	82
I would take part in the study again	10	1	0	0	2	7	90	70
Knee joint aspiration was acceptable	8	1	0	1	1	5	75	63
Leucapheresis was acceptable	8	1	0	0	1	6	88	75
Knee ultrasound was acceptable	11	1	0	0	1	9	91	82
Knee arthroscopy was acceptable	11	1	1	2	3	4	64	36

### Potential efficacy

**Figure 2:**
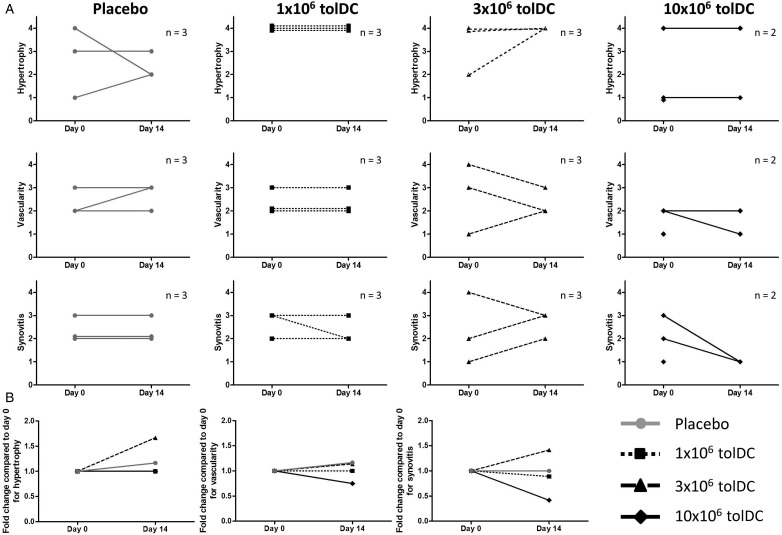
Arthroscopic synovitis scores are presented. Hypertrophy, vascularity and synovitis were scored on a 0–4 scale (17). (A) Individual patient data are illustrated for days 0 and 14 (one patient in the 10×10^6^ tolerogenic dendritic cells (tolDC) cohort declined day 14 arthroscopy). (B) Fold change is shown in hypertrophy, vascularity and synovitis scores compared with day 0. Data are plotted as the mean value for each cohort.

Figure 2 illustrates the exploratory outcome of arthroscopic assessment of target knee synovitis on day 0 and day 14. A 0–4 scale was used to estimate synovial hypertrophy, vascularity and synovitis. One participant in the 10×10^6^ tolDC cohort declined day 14 arthroscopy. There was no evidence for change in synovial hypertrophy with tolDC, improvement being noted in only one control participant. Two of three participants receiving 3×10^6^ tolDC and one of two assessable participants receiving 10×10^6^ tolDC demonstrated improvement in vascularity on day 14, whereas no improvement was seen in six participants receiving 1×10^6^ tolDC or control intervention. Synovitis improved in one of three participants in each of the 1×10^6^ and 3×10^6^ tolDC cohorts and in both assessable patients in the 10×10^6^ tolDC cohorts, but zero of three controls. Notably there was no worsening of any arthroscopic parameter at day 14 in the 10×10^6^ tolDC cohort. Furthermore, although most patients did not report target knee flares, 10 participants received IA glucocorticoid at day 14 for arthroscopically evident synovitis. The exceptions were two participants in the 10×10^6^ tolDC cohort, one of whom declined day 14 arthroscopy due to symptom resolution (participant 10) and the other, participant 12, had near complete resolution of synovitis at arthroscopy. Both remained asymptomatic and declined day 91 arthroscopies. Interestingly, participant 8, whose cells failed release criteria, also did not require IA glucocorticoid on day 14; day 91 arthroscopy revealed stable hypertrophy and vascularity but mild worsening of synovitis. There was no consistent trend in DAS28 or HAQ scores across the dosing cohorts (data not shown), nor in peripheral blood T-cell phenotype or serum cytokine levels (see online supplementary figures S5 and S6).

## Discussion

The primary purpose of this unblinded Phase I trial was to assess the safety of autologous tolDC therapy. We consequently designed our trial to provide a robust signal of worsening synovitis and a means to address this if it occurred. IA administration served both purposes: a tolDC-induced flare should have caused a rapid and significant increase in target knee synovitis, in which case the joint could be irrigated and treated with local glucocorticoid. We estimated 5 days as a likely time course, because pathogenic T cells are enriched in an inflamed joint^18–20^ and could potentially be activated if tolDC were unstable. No flare occurred within this timeframe although four episodes of target knee or systemic synovitis were recorded between days 7 and 10, in participants receiving 1×10^6^ or 3×10^6^ tolDC. Furthermore, at day 14, local glucocorticoid was administered for arthroscopic synovitis in all patients in the 1×10^6^ and 3×10^6^ tolDC cohorts as well as all three controls. In contrast, two of three participants who received 10×10^6^ tolDC did not require local glucocorticoid throughout the study. Therefore, we cannot state unequivocally that tolDC therapy is safe but it is possible that most participants in this small trial received a subtherapeutic dose of tolDC, the ‘flares’ and arthroscopic synovitis on day 14 reflecting the natural history of synovitis following joint irrigation.

Participant 3, with refractory RA, suffered two SAEs. The first was a disease flare 10 weeks after tolDC treatment. Adalimumab was switched to tocilizumab but pneumonia developed 14 days later. Neither SAE was felt related to tolDC therapy. This participant also suffered a generalised RA flare on day +9, suggesting they had unstable disease. Skin psoriasis was reported as stable at baseline in patients 4 and 10 and, therefore, minor worsening on days 6 and 62 was deemed potentially attributable to tolDC. The only infections recorded were two wound infections related to arthroscopy ports, two upper respiratory tract infections, two episodes of rhinorrhoea and one of folliculitis. All AEs were categorised as mild or moderate, with no dose–response.

Each tolDC product was subject to QC assessment. Purity, surface phenotype and cytokine production were based on characteristics which we have consistently observed to distinguish tolDC from mature DCs. Because of a necessarily narrow time window following completion of manufacture (approximately 3 h), some data were unavailable at the time of administration. Sterility of the administered product, and cytokine production, only became available later (secondary release criteria). Only one product failed to meet primary release criteria, with CD86 expression modestly above the specified limit. Despite the intensive nature of the protocol, 91% of participants rated their experience as acceptable. Arthroscopy itself was rated least favourably, but most participants totally (4/11) or partially (3/11) rated it as acceptable. About 90% would participate again in a similar study.

On the basis of this small, unblinded, Phase I trial we believe that tolDC therapy is safe and worthy of further investigation. This conclusion is based on the absence of protocol-defined target knee flares and on anecdotal evidence of improvement in participants in the highest dose cohort. There were three knee flares recorded 7–10 days post-tolDC administration but these occurred in the lower dose cohorts and are therefore more likely to reflect the natural history of knee synovitis following joint irrigation. Because there were no prior reports of tolDC administration in participants with inflammatory arthritis, our dosing regimen was based on cancer strategies. In those scenarios, however, mature DCs boost an anti-tumour immune response and extrapolation to tolerance induction is not necessarily appropriate. Indeed, extrapolation from our prior work in CIA would have predicted a higher therapeutic dose. In contrast, in a recently published study in RA, 1×10^6^ and 5×10^6^ autologous modified DCs loaded with citrullinated peptide antigens demonstrated possible clinical benefit and biological activity.^13^ However, those cells were manufactured by exposure to a nuclear factor kappa-light-chain-enhancer of activated B cells (NF-κB) inhibitor and were administered intradermally. The IA route, while providing a robust safety read-out, may provide a more challenging environment for tolDC to demonstrate efficacy. An important additional question is whether the IA route could provide a systemic effect. No such effect was evident in our study, either clinically or in terms of T-cell modulation. We are currently planning an extension to AuToDeCRA in which 10×10^6^ tolDC will be radiolabelled before IA administration. This will address whether IA tolDC migrate to local lymph nodes, where they could modulate the systemic immune response.

Our protocol enabled treatment of a range of arthritides. Seven participants had RA, three had psoriatic arthritis and two had undifferentiated seronegative arthritis. Although psoriatic arthritis may be considered a disease of the innate immune system, there remains considerable support for an autoimmune aetiology.^21^ Furthermore, some tolDC safety concerns (eg, sterility, potential for proinflammatory cytokine release) are independent of the disease being treated. In fact the patients with possible sustained responses had psoriatic arthritis and seronegative undifferentiated arthritis. This may reflect diagnostic imbalance across dosing cohorts, the only RA patient in the 10×10^6^ tolDC cohort receiving control intervention. Nonetheless, these data emphasise the safety and potential utility of tolDC across a range of arthritides with differing aetiology. Notably, targeting IL-17 is effective in psoriatic arthritis and tolDC deviate T-cells in CIA from IL-17 to IL-10 production.^11^

While ideal for assessing safety, and well-tolerated by participants, IA tolDC administration is an invasive intervention. TolDC have also been administered intradermally in juvenile type 1 diabetes^22^ and intraperitoneally in Crohn's disease.^23^ Intradermal administration provides a more convenient route of administration particularly if, as in our preclinical studies, multiple tolDC administrations are ultimately required for robust efficacy.^11^ Similarly, loading tolDC with autologous SF broadens the target population and obviates the need for tissue-typing, which is generally necessary when loading tolDC with autoantigenic peptides. Nonetheless, joint aspiration is a further invasive procedure and three participants were excluded because SF could not be obtained. Thus, there are advantages and disadvantages to our current protocol. Nonetheless, we believe that AuToDeCRA has defined a safe, and potentially active, dose of tolDC on which to base future work.

## References

[1] Nagy G, van Vollenhoven RF Sustained biologic-free and drug-free remission in rheumatoid arthritis, where are we now? Arthritis Res Ther 2015;17:181 10.1186/s13075-015-0707-1 26235544 PMC4522973

[2] Steinman RM Dendritic cells: understanding immunogenicity. Eur J Immunol 2007;37(Suppl 1):S53–60. 10.1002/eji.200737400 17972346

[3] Diebold SS Determination of T-cell fate by dendritic cells. Immunol Cell Biol 2008;86:389–97. 10.1038/icb.2008.26 18382438

[4] Osorio F, Fuentes C, López MN, et al Role of dendritic cells in the induction of lymphocyte tolerance. Front Immunol 2015;6:535 10.3389/fimmu.2015.00535 26539197 PMC4611163

[5] Hilkens CM, Isaacs JD, Thomson AW Development of dendritic cell-based immunotherapy for autoimmunity. Int Rev Immunol 2010;29:156–83. 10.3109/08830180903281193 20199240

[6] Ganguly D, Haak S, Sisirak V, et al The role of dendritic cells in autoimmunity. Nat Rev Immunol 2013;13:566–77. 10.1038/nri3477 23827956 PMC4160805

[7] Anderson AE, Sayers BL, Haniffa MA, et al Differential regulation of naïve and memory CD4+ T cells by alternatively activated dendritic cells. J Leukoc Biol 2008;84:124–33. 10.1189/jlb.1107744 18430785 PMC2504714

[8] Anderson AE, Swan DJ, Sayers BL, et al LPS activation is required for migratory activity and antigen presentation by tolerogenic dendritic cells. J Leukoc Biol 2009;85:243–50. 10.1189/jlb.0608374 18971286 PMC2700018

[9] Harry RA, Anderson AE, Isaacs JD, et al Generation and characterisation of therapeutic tolerogenic dendritic cells for rheumatoid arthritis. Ann Rheum Dis 2010;69:2042–50. 10.1136/ard.2009.126383 20551157 PMC3002758

[10] Hilkens CM, Isaacs JD Tolerogenic dendritic cell therapy for rheumatoid arthritis: where are we now? Clin Exp Immunol 2013;172:148–57. 10.1111/cei.12038 23574312 PMC3628318

[11] Stoop JN, Harry RA, von Delwig A, et al Therapeutic effect of tolerogenic dendritic cells in established collagen-induced arthritis is associated with a reduction in Th17 responses. Arthritis Rheum 2010;62:3656–65. 10.1002/art.27756 20862679

[12] Ouyang W, Rutz S, Crellin NK, et al Regulation and functions of the IL-10 family of cytokines in inflammation and disease. Annu Rev Immunol 2011;29:71–109. 10.1146/annurev-immunol-031210-101312 21166540

[13] Benham H, Nel HJ, Law SC, et al Citrullinated peptide dendritic cell immunotherapy in HLA risk genotype-positive rheumatoid arthritis patients. Sci Transl Med 2015;7:290ra87 10.1126/scitranslmed.aaa9301 26041704

[14] Tsark EC, Wang W, Teng Y-C, et al Differential MHC class II-mediated presentation of rheumatoid arthritis autoantigens by human dendritic cells and macrophages. J Immunol 2002;169:6625–33. 10.4049/jimmunol.169.11.6625 12444176

[15] Seward RJ, Drouin EE, Steere AC, et al Peptides presented by HLA-DR molecules in synovia of patients with rheumatoid arthritis or antibiotic-refractory Lyme arthritis. Mol Cell Proteomics 2011;10:M110.002477 10.1074/mcp.M110.002477 PMC3047150 21081667

[16] van Beers JJ, Schwarte CM, Stammen-Vogelzangs J, et al The rheumatoid arthritis synovial fluid citrullinome reveals novel citrullinated epitopes in apolipoprotein E, myeloid nuclear differentiation antigen, and β-actin. Arthritis Rheum 2013;65:69–80. 10.1002/art.37720 23044660

[17] af Klint E, Catrina AI, Matt P, et al Evaluation of arthroscopy and macroscopic scoring. Arthritis Res Ther 2009;11:R81 10.1186/ar2714 19490631 PMC2714131

[18] Chabaud M, Durand JM, Buchs N, et al Human interleukin-17: a T cell-derived proinflammatory cytokine produced by the rheumatoid synovium. Arthritis Rheum 1999;42:963–70. 10.1002/1529-0131(199905)42:5<963::AID-ANR15>3.0.CO;2-E 10323452

[19] Shahrara S, Huang Q, Mandelin AM 2nd, et al TH-17 cells in rheumatoid arthritis. Arthritis Res Ther 2008;10:R93 10.1186/ar2477 18710567 PMC2575607

[20] Reynolds G, Gibbon JR, Pratt AG, et al Synovial CD4+ T-cell-derived GM-CSF supports the differentiation of an inflammatory dendritic cell population in rheumatoid arthritis. Ann Rheum Dis 2015 10.1136/annrheumdis-2014-206578 PMC4853576 25923217

[21] FitzGerald O, Haroon M, Giles JT, et al Concepts of pathogenesis in psoriatic arthritis: genotype determines clinical phenotype. Arthritis Res Ther 2015;17:115 10.1186/s13075-015-0640-3 25948071 PMC4422545

[22] Giannoukakis N, Phillips B, Finegold D, et al Phase I (safety) study of autologous tolerogenic dendritic cells in type 1 diabetic patients. Diabetes care 2011;34:2026–32. 10.2337/dc11-0472 21680720 PMC3161299

[23] Jauregui-Amezaga A, Cabezón R, Ramírez-Morros A, et al Intraperitoneal administration of autologous tolerogenic dendritic cells for refractory crohn's disease: a phase I study. J Crohns Colitis 2015;9:1071–8. 10.1093/ecco-jcc/jjv144 26303633

