# Supplementary material for: Autologous tolerogenic dendritic cells for rheumatoid and inflammatory arthritis
Source: Ann Rheum Dis. 2016 Apr 26;76(1):227–34. doi: 10.1136/annrheumdis-2015-208456 (PMC5264217; doi:10.1136/annrheumdis-2015-208456)
Supplement: Supplementary data [file annrheumdis-2015-208456supp.pdf]

## Supplementary Methods

## tolDC manufacture

Subjects (*Homo sapiens*), over the age of 18 with active inflammatory arthritis, including an inflamed knee joint were screened for mandatory infectious markers in accordance with the Human Tissue Regulations 2007 and deemed to be negative for all markers. In addition, synovial fluid (SF) was aspirated from an inflamed knee joint under High Resolution Ultrasound. SF was treated with heparin (1IU/ml) and hyaluronidase (10IU/ml) at 37°C for 30 minutes and centrifuged to remove cells and debris, prior to storage at-80°C until required.

Patients underwent a routine leukapheresis procedure to harvest white blood cells (Ward 36, Freeman Hospital). Donations were transported at ambient temperature and analysed using the Sysmex XE-2100 haematology system to confirm the presence of ≥$1x10^{10}$ nucleated cells before further processing. Peripheral blood mononuclear cells were isolated by density gradient centrifugation on a Cobe 2991 cell processor followed by positive selection using anti-CD14 microbeads and the CliniMACS system (Miltenyi Biotec, Surrey), according to the manufacturer's instructions.

Cells were held at 2-8°C in PBS/1 mM EDTA/0.5% human serum albumin (HAS) until CD14+ cell numbers were confirmed as ≥$5x10^7$ using the Sysmex XE-2100 then immediately cultured for 7 days in a closed bag system (100ml cell expansion bag; Miltenyi Biotec) in the presence of cytokines and immunosuppressive drugs. $1x10^8$ cells were seeded at a density of $2x10^6$ cells/ml in CellGro DC® serum free culture medium (CellGenix, Freiburg, Germany) supplemented with human recombinant IL-4 (1000IU/ml; CellGenix) and human recombinant GM-CSF (1000IU/ml; Bayer, Seattle) to induce dendritic cell differentiation. After 3 days the culture volume was doubled by adding fresh pre-warmed CellGro DC® culture medium and replenishment of cytokines to a final concentration of 1000IU/ml. Cells were tolerised by the addition of dexamethasone ($10^{-6}$M; Organon) on day 3 and further addition of dexamethasone ($10^{-6}$M) plus the active form of vitamin D3; calcitriol ($10^{-10}$M, Tocris) on day 6. At the same time, cells were also loaded with autoantigen in the form of autologous SF (≤10% v/v) in the presence of the synthetic TLR4 ligand; monophosphoryl lipid A (MPLA; 1µg/ml, Avanti Polar Lipids, Alabama, USA) for the final 20 hours of culture to induce a stable semi-mature phenotype.

All processes were performed in a dedicated clean room under GMP conditions following Standard Operating Procedures. Cells were cultured at 37°C in the presence of 5% $CO_2$. All manipulations were performed using culture media and reagents pre-warmed to ambient temperature. Visual inspection of the culture on days 3 and 6 assessed signs of bacterial contamination. Samples were also removed from the culture on day 0 and 3 and tested for

aerobic and anaerobic contamination using the BacT/Alert® system (Biomerieux, Basingstoke).

On day 7, the day of tolDC administration, cells were washed 4 times in saline 0.5% HAS in 50 ml centrifuge tubes to remove all traces of unbound reagents. Prior to release tolDC had to satisfy the following quality control criteria: >70% viability determined by trypan blue exclusion; >90% purity determined by surface expression of both CD11c and MHC II; CD86 expression MFI>1500 and CD83 expression MFI>350. Samples taken from starting products had to show no contamination with bacterial (in CD14+ monocytes and SF samples) or fungal growth (in CD14+ monocytes samples) after a 5-day test. Day 3 tolDC culture samples had to show no contamination with bacterial or fungal growth after a 3-day test.

Depending on the prescribed dose, up to $1x10^7$ cells were resuspended in a volume of 8-10ml saline 1% HAS in a sealed cryocyte bag (CS50, Origen) The product was transported to the clinic at ambient temperature and administered to the patient arthroscopically within 2 hours of tolDC release.

Secondary release criteria, available retrospectively, demonstrated that the final tolDC product passed Eu.Pharma sterility tests. Levels of secreted cytokines were measured by ELISA; IL-10 levels were shown to be >1000pg/ml and IL-12p70 levels <50pg/ml, demonstrating the tolDC had an anti-inflammatory phenotype.

## Biomarker analyses

*Patient material*

Peripheral blood was obtained from each patient at baseline (day 0) and on days 14 and day 91. PBMCs were isolated by density centrifugation on Lymphoprep (Axis-Shield Diagnostics, Dundee, UK) and frozen at −80°C until use. Serum was isolated and frozen at −80°C until use.

*Flow cytometry*

PBMC were thawed and IFN- γ, IL-17A, IL-10 and FoxP3 expression assessed using flow cytometry. Intracellular IFN-γ and IL-17A staining used anti-human IFN-γ-FITC (4S.B3; BD Biosciences, San Jose, CA, USA) and anti-human IL-17A-eFluor 660 (eBio64DEC17; eBioscience, San Diego, CA, USA) antibodies. PBMC were stimulated with PMA (5 ng/ml) and ionomycin (500 ng/ml), and after 1 h, Brefeldin A (10 µg/ml) was added for an additional 3 h. Cells were harvested, stained with Zombie Aqua (BioLegend, San Diego, CA, USA) to assess cell viability, surface-stained with anti-human CD3-PE (UCHT1; BD Biosciences), and then fixed using fixation/permeabilisation buffer (eBioscience). Cells were permeabilised using permeabilisation buffer (eBioscience). To reduce background staining, cells were blocked with 2% mouse serum (Sigma-Aldrich, Dorset, UK) for 15 min prior to addition of IFN- γ and IL-17A antibodies and anti-human CD4PerCP (SK3; BD Biosciences). Cells were incubated at 4°C for 30 minutes, centrifuged, and resuspended in staining buffer (PBS supplemented with

0.5% BSA, 2 mM EDTA, and 0.01% sodium azide). Intracellular forkhead box P3 (FoxP3) was detected using an anti- human FoxP3-Alexa Fluor 647 antibody (206D; BioLegend) in combination with anti-human CD3-FITC (HIT3a; BD Biosciences) and anti-human CD4-PerCP antibodies using the FoxP3 staining buffer set (eBioscience). Human IgG (Grifols, Los Angeles, CA, USA) was added with antibodies to prevent Fc[illegible]c binding and cell viability was assessed using Zombie Aqua prior to cell fixation. To assess IL-10 production, PBMC were stimulated with T cell CD3/CD28 expander beads (1:1, Dynal, Invitrogen, Paisley, UK) for 18 h, and IL-10 secretion was measured using an IL-10-PE secretion assay (Miltenyi Biotec, Bergisch Gladbach, Germany). Cells were also surface-stained with anti-human CD3-APC (UCHT1; BioLegend) and anti-human CD4-APC eFluor 780 (SK3; eBioscience) antibodies and cell viability was assessed by the addition of DAPI (Invitrogen) prior to acquisition. All samples were acquired on a BD Biosciences FACSCanto II and were analysed using FlowJo (Tree Star, Ashland, OR, USA).

*Serum cytokine measurement*

Serum IFN-γ, IL-10, IL-12p70, IL-13, IL-1β, IL-2, IL-4, IL-6, IL-8, TNF-α, and IL-17A were measured using a highly sensitive, validated electrochemiluminescence detection system (Meso Scale Discovery, Gaithersberg, Massachusetts, USA).
