# Supplementary material for: Autologous tolerogenic dendritic cells for rheumatoid and inflammatory arthritis
Source: Ann Rheum Dis. 2016 Apr 26;76(1):227–34. doi: 10.1136/annrheumdis-2015-208456 (PMC5264217; doi:10.1136/annrheumdis-2015-208456)
Supplement: Supplementary data [file annrheumdis-2015-208456supp2.pdf]

Supplementary Figures:

Supplementary Figure S1. Knee Assessment Questionnaire

Thank you for taking the time to fill in this questionnaire.

Please answer the questions below by circling the number where you feel your knee symptoms are today:

0 no symptoms

1 slight symptoms

2 mild symptoms

3 moderate symptoms

4 severe symptoms

5 worst imaginable symptoms

1. Where is your knee pain today?

   0 1 2 3 4 5

2. Where is your knee swelling today?

   0 1 2 3 4 5

3. Where is your knee stiffness today?

   0 1 2 3 4 5

4. Do you think that you are having a 'flare' in your knee today?

   0 1 2 3 4 5

Finally, do you think that your knee has deteriorated overall since the baseline visit, before the treatment was given? Please answer yes or no!

Thank you

Supplementary Figure S2. Physician VAS for knee activity

Please place a vertical mark through the line where 0 = no disease activity and 10= worst possible disease activity for selling, temperature, range of movement, erythema and overall disease activity:

1. Swelling

0 ____ 10

2. Temperature

0 ____ 10

3. Range of Movement

0 ____ 10

4. Erythema

0 ____ 10

5. Overall disease activity

0 ____ 10

Supplementary Figure S3. Participant Acceptability Questionnaire

Please circle the number that you feel applies best to the statements below:

1= totally disagree

2= partially disagree

3 = neither agree nor disagee

4 = partially agree

5= totally agree

1. Taking part in the study was convenient

1 2 3 4 5

2. The overall study was acceptable

1 2 3 4 5

3. I would take part in the study again

1 2 3 4 5

4. I found the knee joint aspiration acceptable

1 2 3 4 5

5. I found leukapheresis acceptable

1 2 3 4 5

6. I found the knee ultrasound scan acceptable

1 2 3 4 5

7. I found the arthroscopy (camera examination of the knee joint) acceptable

1 2 3 4 5

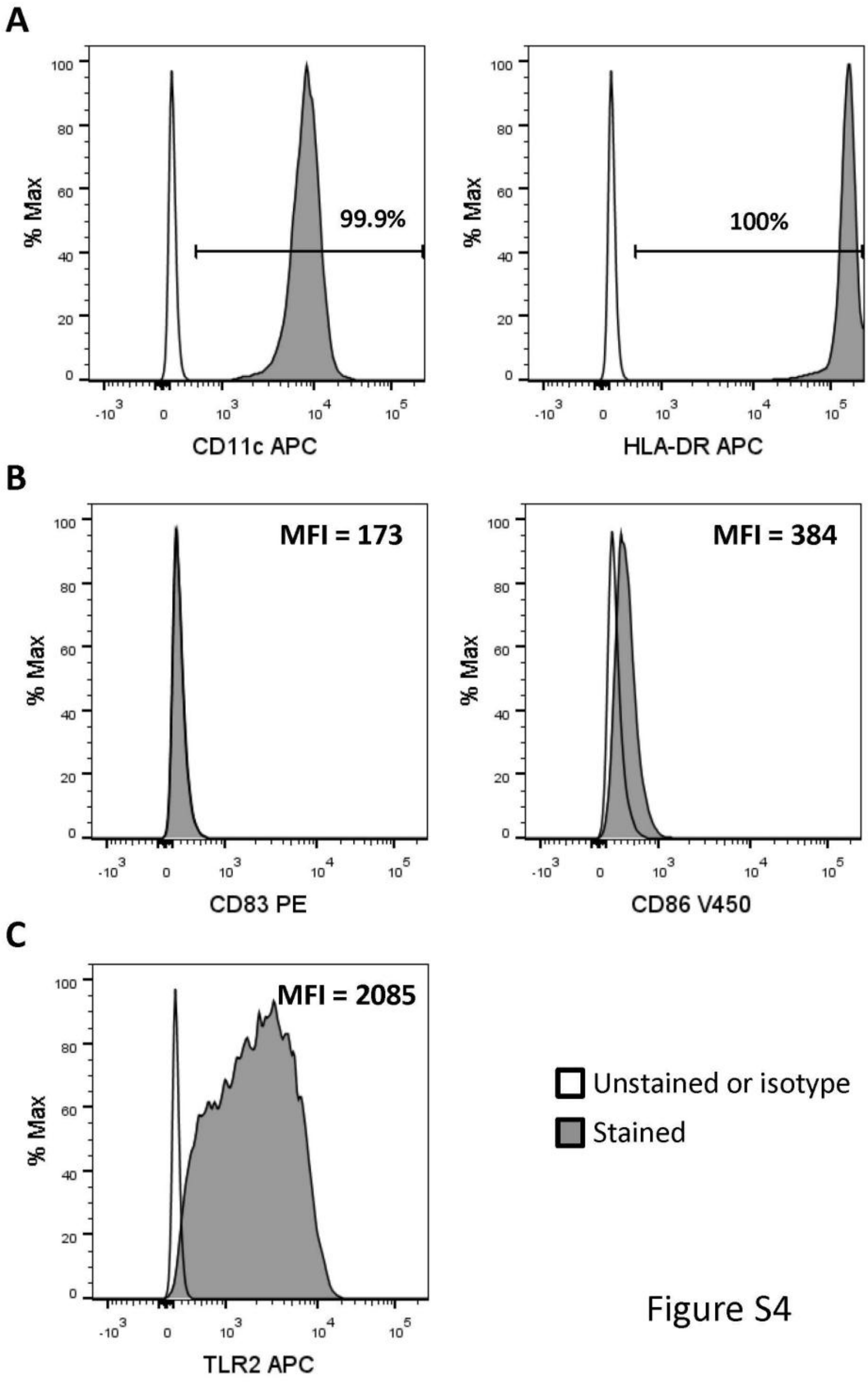
A
% Max
99.9%
CD11c APC
100%
HLA-DR APC
B
MFI = 173
CD83 PE
MFI = 384
CD86 V450
C
MFI = 2085
TLR2 APC
Unstained or isotype
Stained

Figure S4

Figure 5

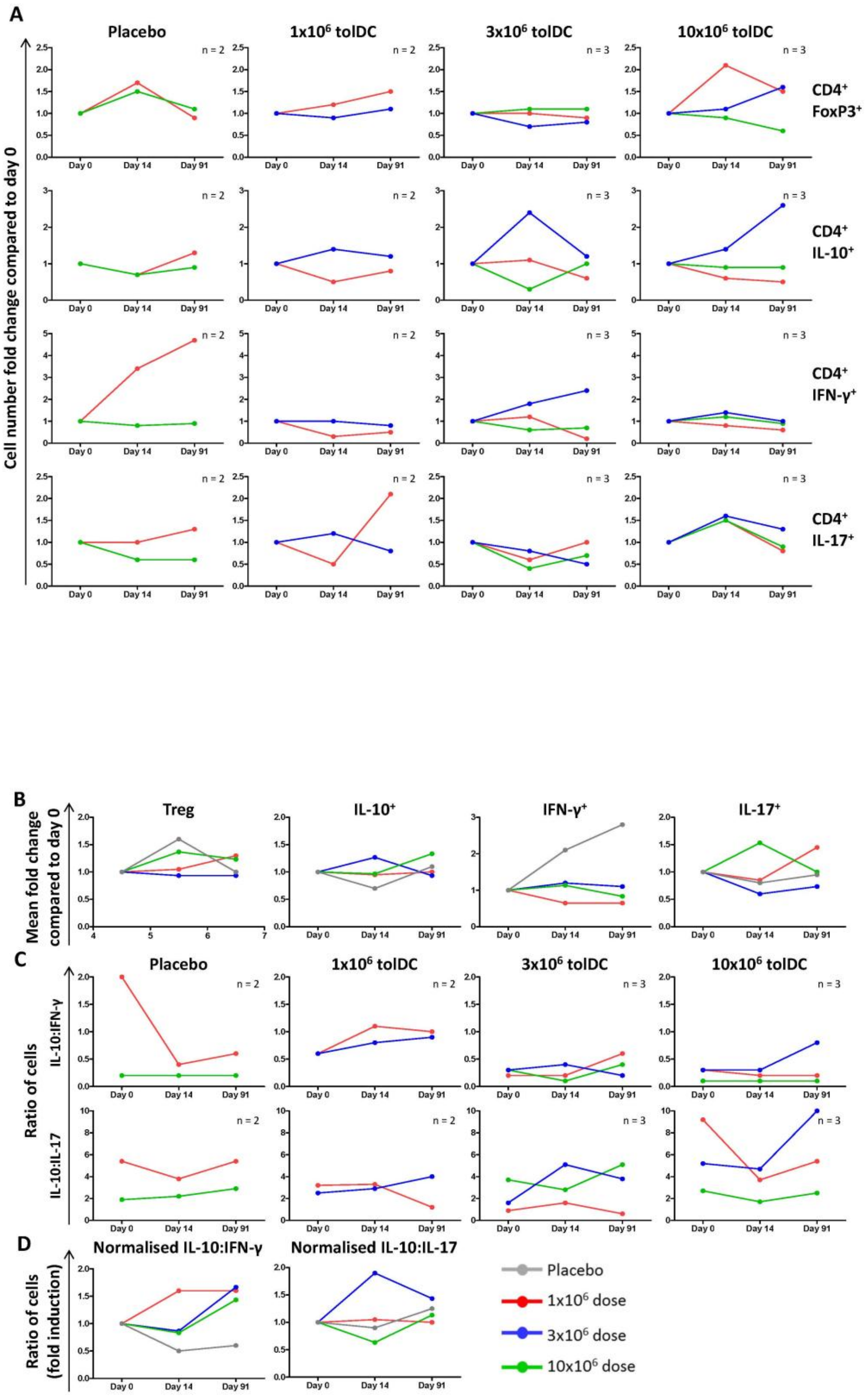
A
Placebo
1x10^6 tolDC
3x10^6 tolDC
10x10^6 tolDC
Cell number fold change compared to day 0
CD4+ FoxP3+
CD4+ IL-10+
CD4+ IFN-γ+
CD4+ IL-17+
n = 2
n = 3
Day 0
Day 14
Day 91
B
Mean fold change compared to day 0
Treg
IL-10+
IFN-γ+
IL-17+
C
Ratio of cells
IL-10:IFN-γ
IL-10:IL-17
D
Ratio of cells (fold induction)
Normalised IL-10:IFN-γ
Normalised IL-10:IL-17
Placebo
1x10^6 dose
3x10^6 dose
10x10^6 dose

Figure S6

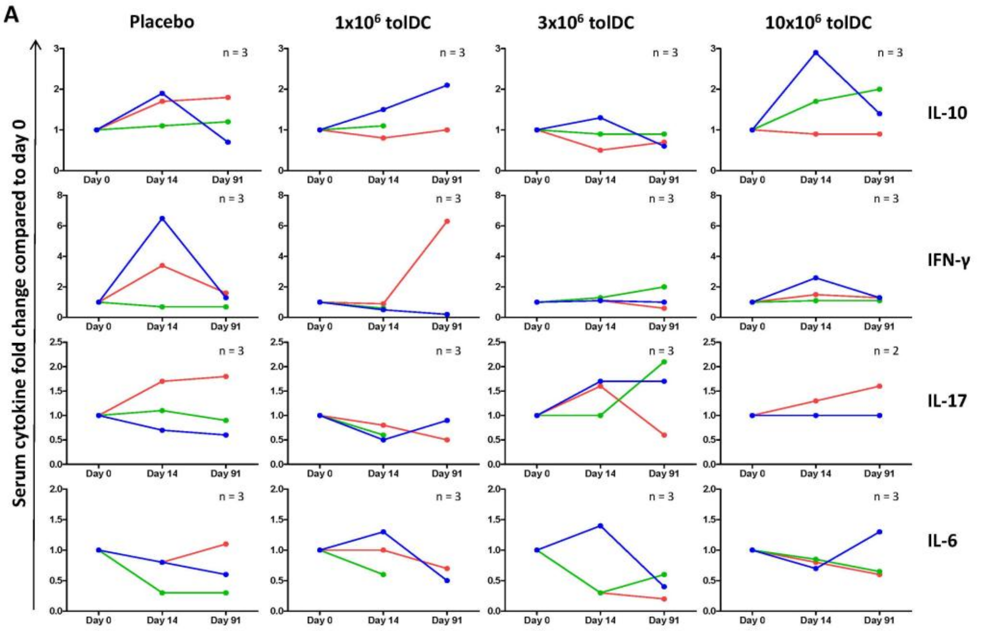
A
Placebo
1x10⁶ tolDC
3x10⁶ tolDC
10x10⁶ tolDC
IL-10
IFN-γ
IL-17
IL-6
Serum cytokine fold change compared to day 0
n = 3
n = 2
Day 0
Day 14
Day 91

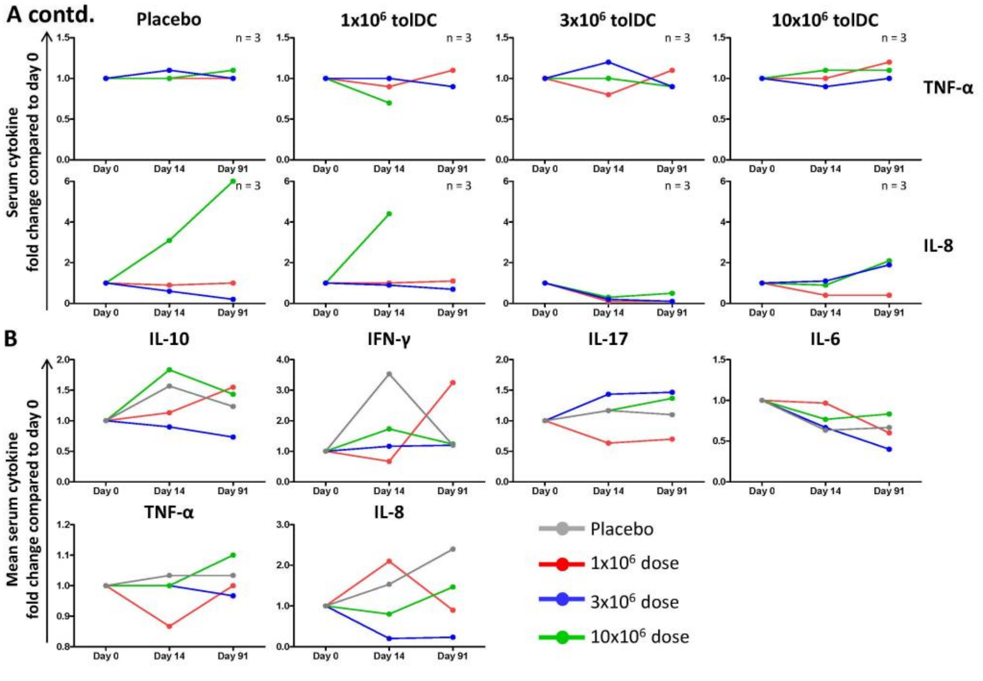
A contd.
Placebo
1x10⁶ tolDC
3x10⁶ tolDC
10x10⁶ tolDC
TNF-α
IL-8
Serum cytokine fold change compared to day 0
n = 3
B
IL-10
IFN-γ
IL-17
IL-6
TNF-α
IL-8
Mean serum cytokine fold change compared to day 0
Day 0
Day 14
Day 91
Placebo
1x10⁶ dose
3x10⁶ dose
10x10⁶ dose

C

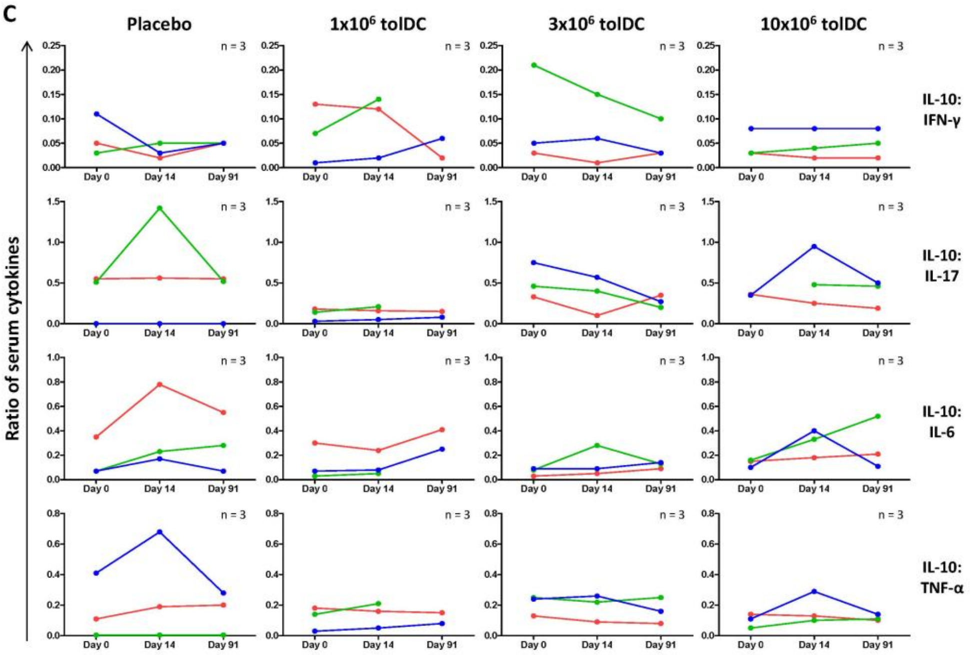
Placebo
1x10⁶ tolDC
3x10⁶ tolDC
10x10⁶ tolDC
n = 3
IL-10:
IFN-γ
IL-10:
IL-17
IL-10:
IL-6
IL-10:
TNF-α
Ratio of serum cytokines
Day 0
Day 14
Day 91

C contd.

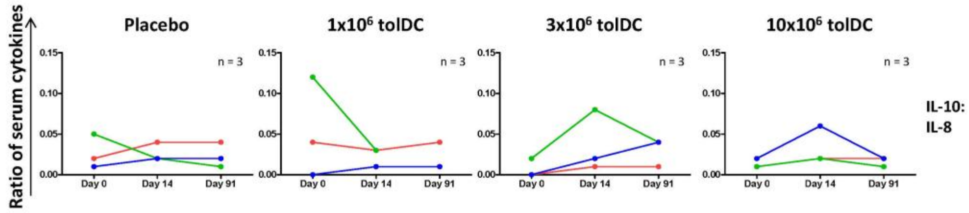
Placebo
1x10⁶ tolDC
3x10⁶ tolDC
10x10⁶ tolDC
n = 3
IL-10:
IL-8
Ratio of serum cytokines
Day 0
Day 14
Day 91

D

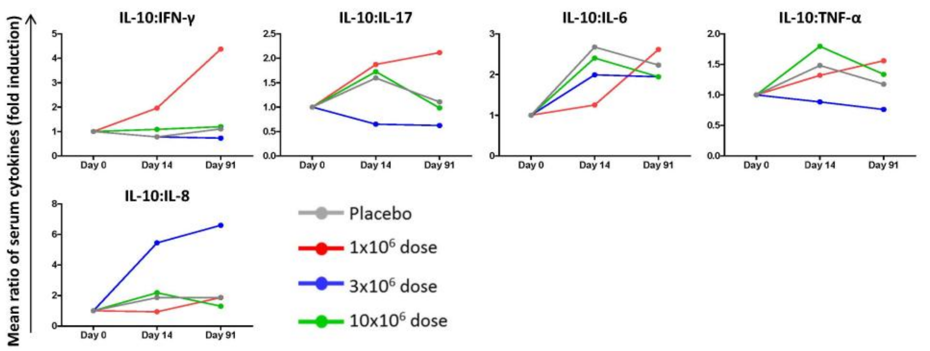
IL-10:IFN-γ
IL-10:IL-17
IL-10:IL-6
IL-10:TNF-α
IL-10:IL-8
Mean ratio of serum cytokines (fold induction)
Day 0
Day 14
Day 91
Placebo
1x10⁶ dose
3x10⁶ dose
10x10⁶ dose

Supplementary Table S1. AuToDeCRA exclusion criteria

Serious or unstable co-morbidity
History of malignancy (except treated basal cell carcinoma of skin)
Known active infection at screening or at baseline (except fungal nail infection)
Infection requiring hospitalization or IV antibiotics within 6 weeks of baseline
Immunization with a live vaccine within 6 weeks of baseline
History of recurrent or chronic infection
History of and/or positive testing for the mandatory markers of hepatitis B or C, syphilis, HIV, CMV or HTLV-1/2 infections (requirements for GMP)
Standard haematological and biochemical exclusions
Pregnancy, planned pregnancy and breast feeding
